# Weight Status, Autonomic Function, and Systemic Inflammation in Children with Obstructive Sleep Apnea

**DOI:** 10.3390/ijms25168951

**Published:** 2024-08-16

**Authors:** Hai-Hua Chuang, Chung-Guei Huang, Jen-Fu Hsu, Li-Pang Chuang, Yu-Shu Huang, Hsueh-Yu Li, Li-Ang Lee

**Affiliations:** 1 Department of Family Medicine, Metabolism and Obesity Institute, Sleep Center, Chang Gung Memorial Hospital, Taipei and Linkou Branches, Taoyuan 33305, Taiwan; chhaihua@gmail.com; 2 School of Medicine, College of Medicine, Chang Gung University, Taoyuan 33302, Taiwan; jeff0724@gmail.com (J.-F.H.); lpchuang1678@yahoo.com.tw (L.-P.C.); yushuhuang1212@gmail.com (Y.-S.H.); hyli38@cgmh.org.tw (H.-Y.L.); 3 School of Medicine, College of Life Science and Medicine, National Tsing Hua University, Hsinchu 300044, Taiwan; 4 Department of Community Medicine, Cathay General Hospital, Taipei 106, Taiwan; 5 Department of Laboratory Medicine, Chang Gung Memorial Hospital, Linkou Branch, Taoyuan 33305, Taiwan; joyce@cgmh.org.tw; 6 Department of Medical Biotechnology and Laboratory Science, Graduate Institute of Biomedical Sciences, Chang Gung University, Taoyuan 33302, Taiwan; 7 Department of Pediatrics, Chang Gung Memorial Hospital, Linkou Branch, Taoyuan 33305, Taiwan; 8 Department of Pulmonary and Critical Care Medicine, Sleep Center, Chang Gung Memorial Hospital, Linkou Branch, Taoyuan 33305, Taiwan; 9 Department of Child Psychiatry, Sleep Center, Chang Gung Memorial Hospital, Linkou Branch, Taoyuan 33305, Taiwan; 10 Department of Otorhinolaryngology—Head and Neck Surgery, Metabolism and Obesity Institute, Sleep Center, Chang Gung Memorial Hospital, Linkou Branch, Taoyuan 33305, Taiwan

**Keywords:** autonomic function, heart-rate variability, interleukin, mediation and moderation, obesity, obstructive sleep apnea, systemic inflammation

## Abstract

Children with obstructive sleep apnea (OSA) frequently experience chronic low-grade systemic inflammation, with the inflammasome playing a central role in OSA. This cross-sectional study evaluated the relationship between weight status, autonomic function, and systemic inflammation in a cohort of 55 children with OSA, predominantly boys (78%) with an average age of 7.4 ± 2.2 years and an apnea-hypopnea index of 14.12 ± 17.05 events/hour. Measurements were taken of body mass index (BMI), sleep heart-rate variability, morning circulatory levels of interleukin-1β, interleukin-1 receptor antagonist, and interleukin-6, and tumor necrosis factor-α, anthropometry, and polysomnography. Multiple linear regression modeling showed that an apnea-hypopnea index was significantly associated with BMI, the standard deviation of successive differences between normal-to-normal intervals during N3 sleep, and the proportion of normal-to-normal interval pairs differing by more than 50 ms during rapid-eye-movement sleep. A moderated mediation model revealed that interleukin-1 receptor antagonist levels mediated the association between BMI and interleukin-6 levels, with sympathovagal balance during N3 sleep and minimum blood oxygen saturation further moderating these relationships. This study highlights the complex relationships between BMI, polysomnographic parameters, sleep heart-rate-variability metrics, and inflammatory markers in children with OSA, underlining the importance of weight management in this context.

## 1. Introduction

Obstructive sleep apnea (OSA) is a prevalent sleep disorder among children, affecting at least 1.2% globally, with the prevalence showing an increasing trend over the past decade [1]. Characterized by recurrent hypopneas and apneas due to upper airway collapse, OSA leads to intermittent hypoxemia, autonomic fluctuations, and sleep fragmentation [2]. Key risk factors for pediatric OSA include obesity, male gender, hypertrophy of the tonsils and adenoids, and persistent snoring [3]. Despite its imperfections, the apnea-hypopnea index (AHI) remains the most extensively studied metric for assessing OSA severity [4], significantly correlating with adenoid grade, tonsil size, body mass index (BMI) z-score, and neck circumference [5,6]. If not adequately addressed, OSA in children can lead to a range of serious health issues, including autonomic dysfunction, systemic inflammation, hypertension, behavioral problems, neurocognitive impairments, and non-alcoholic fatty liver disease [7,8,9,10,11,12]. This highlights the critical importance of understanding the underlying pathophysiology, ensuring early diagnosis, and implementing effective management to prevent these potential complications. Notably, obesity, a common risk factor, shares several confounding factors and complications with OSA, emphasizing the need for integrated treatment approaches.

In children with OSA, disruptions such as sleep fragmentation, arousal, and hypoxemia can elevate sympathetic nervous system activity [13]. Additionally, children with obesity are often found to exhibit cardiac autonomic dysfunction, characterized by a predominance of sympathetic activity [14]. This interrelation makes the study of obesity’s impact on autonomic dysfunction particularly pertinent. Heart-rate variability (HRV) is recognized as a valuable indicator of cardiovascular fluctuations and a promising marker for assessing autonomic function and diagnosing pathological states [15]. HRV is primarily a reflection of indirect underlying pathophysiological processes—whether causal, mediating, or reactive—and is widely used as a biomarker across various health conditions [16]. Both time-domain and frequency-domain HRV analyses conducted on electrocardiograms are crucial for diagnosing diverse clinical and functional conditions [17]. In children with OSA, HRV metrics across all sleep stages indicate increased sympathetic nervous system activity [7]. However, sleep stage-specific HRV measurements show a significant reduction in parasympathetic nervous system activity in children with OSA [18]. Furthermore, the relationships between sleep autonomic conditions and OSA-related complications, such as systemic inflammation, are not well understood in the pediatric population. In adults with OSA, the inflammatory response has been shown to influence HRV [19]. Thus, further investigations into the links between obesity, cardiac autonomic function, and systemic inflammation in this population are warranted.

In animal models, intermittent hypoxia drives toll-like receptor 4/nuclear factor κ-light-chain-enhancer of activated B cells and nucleotide-binding oligomerization domain-like receptor 3 (NLRP3)-signaling pathways, leading to the upregulation and secretion of cytokines interleukin (IL)-1β and IL-6 [20]. However, in humans with OSA, increased oxidative stress appears to induce the release of IL-1β, IL-6, and tumor necrosis factor-α (TNF-α) through mechanisms other than the activation of the NLRP3 inflammasome [21]. Notably, the increasing expression of the NLRP3 inflammasome is a predominant factor in the pathogenesis of obesity-associated adipose tissue inflammation [22]. The production of IL-1 receptor antagonist (IL-1RA), IL-6, and TNF-α by monocytes and macrophages is primarily regulated by adiponectin [23].

Our prior research has demonstrated that serum levels of IL-6 are associated with both the severity of OSA and weight status [24], and that changes in IL-1RA levels independently correlate with changes in the BMI z-score following treatment for pediatric OSA [8]. However, no significant roles were observed for IL-1β and TNF-α in our children with OSA and obesity.

Both OSA and obesity can induce systemic inflammation via hypoxia-inducible factor-1 [25,26] and nuclear factor κB [27,28] pathways, as well as the NLRP3 inflammasome [29,30]. The NLRP3 inflammasome is also linked to the regulation of autonomic function [31]. These previous findings laid the groundwork for investigating the relationship between systemic inflammation and cardiac autonomic function in children with OSA. However, to the best of our knowledge, there is no literature reporting these relationships in pediatric participants with OSA. Therefore, understanding the interactions between weight status, autonomic function, and systemic inflammation is crucial for developing targeted interventions for pediatric OSA. This study aimed to investigate the relationship of weight status in children with OSA with HRV and systemic inflammation, providing new insights into the multifaceted role of obesity in pediatric OSA. The null hypothesis of this study was that weight status was not related to autonomic function or systemic inflammation in this context.

## 2. Results

### 2.1. Participant Characteristics, HRV Metrics, and Systemic Inflammatory Markers

**Figure 1:**
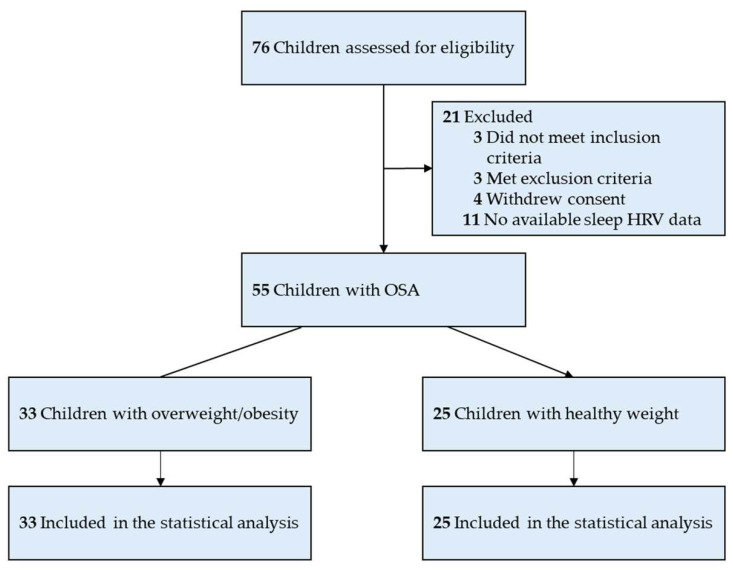
Flowchart of the present study.

The eligibility assessment began with 76 children (Figure 1). Ten candidates were not recruited: three did not meet the inclusion criteria, three met the exclusion criteria, and four rescinded consent. Furthermore, 11 participants were not included in the present study due to no accessible sleep HRV data, as their examinations were performed using different systems at other sleep centers. The remaining cohort comprised 43 boys (78%) and 12 girls (22%), averaging 7.4 ± 2.2 years of age (range, 5–12), with a mean BMI of 19.43 ± 5.55 kg/m^2^, a BMI z-score of 0.680 ± 2.109, and an AHI of 14.12 ± 17.05 events/hour (Table 1).

Participants were categorized into two subgroups based on BMI z-scores: an “overweight/obesity” group (≥1.04) and a “healthy weight” group (<1.04) [32]. Within this group, 30 children (55%) were categorized as overweight/obese, and 25 (45%) were deemed of healthy weight. The subgroup of children with OSA and overweight/obesity had significantly higher averages in age, BMI, BMI z-score, AHI, rapid eye movement (REM)-AHI, and arousal index (ArI) than their healthy-weight counterparts with OSA. The observed covariance matrices of the proportions of the four sleep stages were not equal across the two groups, as indicated by Box’s test (*p* < 0.001). Therefore, Pillai’s trace value was used, resulting in 0.13 (*p* = 0.15) from the one-way multivariate analysis of variance (MANOVA). Additionally, a higher proportion of the N1 sleep stage was observed in the overweight/obesity group compared to the healthy weight group.

Table 2 presents the distribution of HRV metrics across different sleep stages between the overweight/obese and healthy weight groups. The differences in HRV metrics between the groups were not statistically significant. Additionally, the systemic inflammatory markers, including levels of IL-1β, IL-1RA, IL-6, and TNF-α (Table 1), were comparable between the overweight/obesity and healthy weight groups, suggesting similar inflammatory profiles regardless of weight status.

### 2.2. Associations of Polysomnographic Parameters, Weight Status, HRV Metrics, and Systemic Inflammatory Markers

**Figure 2:**
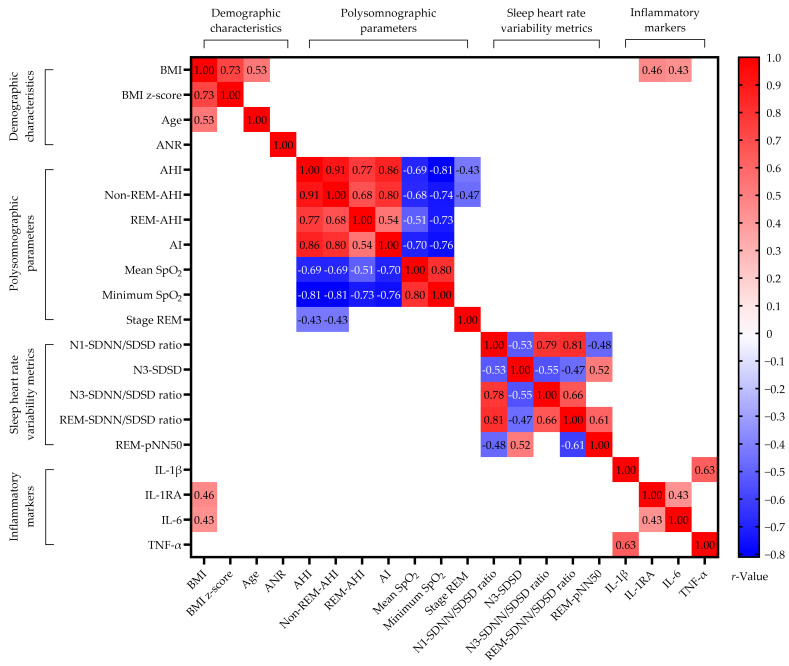
Significant associations across important variables of weight status, polysomnographic parameters, heart-rate-variability metrics, and systemic inflammatory markers. Abbreviations: AHI: apnea-hypopnea index; AI: apnea index; ANR: adenoidal-nasopharyngeal ratio; BMI: body mass index; IL: interleukin; IL-1RA: IL-1 receptor antagonist; pNN50: proportion of N-N interval pairs differing by more than 50 ms; REM: rapid eye movement; SDNN: standard deviation of all normal-to-normal intervals; SDSD: standard deviation of successive differences between normal-to-normal intervals; SpO_2_: blood oxygen saturation; TNF: tumor necrosis factor. Data are summarized as correlation coefficients. Blank spaces mean two-sided adjusted *p*-values ≥ 0.05.

Significant correlations between important variables of weight status, demographic variables, polysomnographic parameters, HRV metrics, and systemic inflammatory biomarkers are depicted in Figure 2. The analysis often showed strong intra-group relationships among variables, and the Bonferroni correction was used to reduce the Type I error due to multiple comparisons. This section specifically emphasizes notable associations related to BMI since the BMI z-score demonstrated weaker correlations compared to other variables. Furthermore, the majority of analyzed variables were associated with age. Therefore, BMI was preferred as the core dependent variable in this study.

AHI was positively correlated with the apnea index (AI) and negatively associated with mean peripheral oxygen saturation (SpO_2_) and minimum SpO_2_. Multivariable linear regression models, employing a forward selection process, identified significant relationships between AHI and BMI (β = 1.21, 95% CI [0.33–2.08], *p* = 0.01, variance inflation factor [VIF] = 1.03), the standard deviation of successive differences between normal-to-normal (N-N) intervals (SDSD) during N3 sleep (β = 0.32, 95% CI [0.13–0.52], *p* = 0.002, VIF = 1.36), and the proportion of N-N interval pairs differing by more than 50 ms (pNN50) during REM stage (β = −0.29, 95% CI [−0.53–−0.06], *p* = 0.01, VIF = 1.39), with an adjusted *r*^2^ of 0.29.

Non-REM-AHI was positively associated with REM-AHI and AI, and it was negatively related to mean SpO_2_, minimum SpO_2_, and REM sleep. Multivariable linear regression models revealed significant associations of non-REM-AHI with BMI (β = 0.71, 95% CI [0.04–1.38], *p* = 0.04, VIF = 1.02) and AI (β = 1.40, 95% CI [1.06–1.74], *p* < 0.001, VIF = 1.02), with an adjusted *r*^2^ of 0.66.

REM-AHI was positively related to non-REM-AHI and AI and negatively associated with mean SpO_2_ and minimum SpO_2_. Multivariable linear regression models identified significant relationships between REM-AHI and BMI (β = 099, 95% CI [0.15–1.18], *p* = 0.02, VIF = 1.05), ArI (β = 2.69, 95% CI [1.88–3.51], *p* < 0.001, VIF = 4.38), and N1 sleep stage (β = −1.71, 95% CI [−2.54–−0.88], *p* < 0.001, VIF = 4.46), with an adjusted *r*^2^ of 0.61.

AI was positively related to AHI and negatively associated with mean SpO_2_ and minimum SpO_2_. Multivariable linear regression models failed to construct a significant model using demographic characteristics, sleep HRV metrics, and inflammatory markers.

Mean SpO_2_ was positively correlated with minimum SpO_2_ and negatively associated with AHI and AI. After multivariate linear regression adjustments, mean SpO_2_ was significantly related to age (β = −0.22, 95% CI [−0.42–−0.02], *p* = 0.03, VIF = 1.40), the standard deviation of all N-N intervals (SDNN)/SDSD during N1 sleep (β = 2.34, 95% CI [1.22–3.46], *p* < 0.001, VIF = 2.97), and the SDNN/SDSD during REM sleep (β = −1.14, 95% CI [−1.90–−0.38], *p* = 0.004, VIF = 3.62), with an adjusted *r*^2^ of 0.38.

Minimum SpO_2_ was positively correlated with mean SpO_2_ and negatively associated with AHI and AI. Multivariable linear regression models revealed significant relationships between minimum SpO_2_ and BMI (β = −0.60, 95% CI [−1.00–−0.19], *p* = 0.01, VIF = 1.00), with an adjusted *r*^2^ of 0.15.

BMI was positively correlated with age and levels of IL-1RA and IL-6. Multivariable linear regression models identified significant relationships between BMI and the SDNN/SDSD ratio during REM sleep (β = 1.98, 95% CI, 0.53–3.43, *p* = 0.02, VIF = 1.34), the proportion of REM stage (β = −0.23, 95% CI, −0.43–−0.03, *p* = 0.02, VIF = 1.04), and age (β = 0.63, 95% CI, 0.01–1.25, *p* = 0.02, VIF = 1.34), with an adjusted *r*^2^ of 0.41.

BMI z-scores were found to be positively correlated with the AHI. Multivariate linear regression analysis demonstrated that the BMI z-score was independently associated with AHI (β = 0.04, 95% CI, 0.01–0.07, *p* = 0.02, VIF = 1.00), with an adjusted *r*^2^ of 0.08.

IL-1β levels exhibited a positive association with TNF-α levels. Multivariate linear regression analysis confirmed a significant association between IL-1β and TNF-α levels (β = 0.03, 95% CI, 0.02–0.04, *p* < 0.001, VIF = 1.00), with an adjusted *r*^2^ of 0.39.

IL-1RA levels were positively correlated with BMI and IL-6 levels. Multivariate linear regression demonstrated that IL-1RA levels were significantly related to the SDNN/SDSD ratio during N1 sleep (β = 78.24, 95% CI, 19.09–137.38, *p* = 0.01, VIF = 1.00) and adenoidal-nasopharyngeal ratio (ANR) (β = −283.76, 95% CI, −547.51–−20.00, *p* = 0.04, VIF = 1.00), with an adjusted *r*^2^ of 0.19.

IL-6 levels were positively associated with BMI and IL-1RA levels. After multivariate linear regression adjustments, IL-6 levels were significantly associated with BMI (β = 0.22, 95% CI, 0.02–0.41, *p* = 0.03, VIF = 1.27) and IL-1RA (β = 0.01, 95% CI, 0.001–0.02, *p* = 0.04, VIF = 1.27), with an adjusted *r*^2^ of 0.22.

TNF-α levels were positively associated with IL-1β levels. Multivariate linear regression showed significant relationships for TNF-α with IL-1β levels (β = 13.62, 95% CI, 8.79–18.44, *p* < 0.001, VIF = 1.03) and age (β = −1.90, 95% CI, −3.78–−0.03, *p* = 0.047, VIF = 1.03), resulting in an adjusted r^2^ of 0.42.

### 2.3. Mediation and Moderation Analysis of the Relationships between Polysomnographic Parameters, Weight Status, HRV Metrics, and Systemic Inflammatory Markers

**Figure 3:**
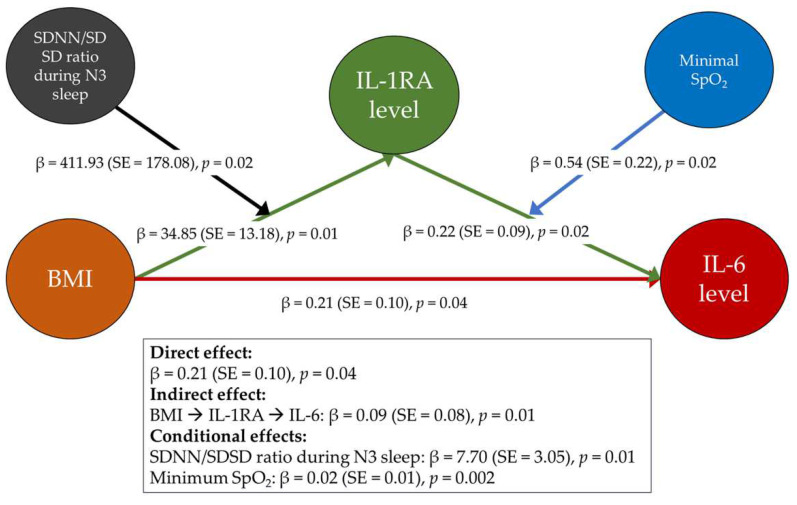
This figure depicts a moderated mediation model with BMI as the independent variable affecting the level of interleukin-6 (IL-6), the dependent variable, mediated by the level of IL-1 receptor antagonist (IL-1RA). Additionally, the standard deviation of all normal-to-normal intervals (SDNN)/standard deviation of successive differences between normal-to-normal intervals (SDSD) ratio during N3 sleep acts as a moderator in the relationship between body mass index (BMI) and IL-1 receptor antagonist (IL-1RA) levels. The minimum blood oxygen saturation (SpO_2_) acts as a moderator in the relationship between IL-1RA and IL-6 levels. Regression coefficients (β) and standard errors (SE) are provided, illustrating both the direct and indirect pathways within the model.

In this section, we focused on the relationship across BMI, IL-1RA, and IL-6 while adding other associated variables of interest. We performed extensive mediation and moderation analyses to achieve this aim and finally found that the IL-1RA level mediated the relationship between BMI and the IL-6 level, while the SDNN/SDSD ratio during N3 sleep moderated the link between BMI and the IL-1RA level, and minimum SpO_2_ moderated the link between IL-1RA and the IL-6 level (direct effect: β = 0.21, standard error = 0.10, *p* = 0.04; indirect effect: β = 0.09, standard error = 0.08, *p* = 0.01) (Figure 3).

## 3. Discussion

Our findings revealed a modest yet significant statistical relationship between increased BMI z-scores and higher AHI values in the studied pediatric population, suggesting that the BMI z-score could be an effective indicator of OSA severity, explaining 8% of the variance. Age- and sex-adjusted BMI z-score is a standard approach for population-based studies [33]. However, our results suggested that BMI, the SDSD during N3 sleep, and the pNN50 during the REM stage were independently related to AHI, explaining 29% of the variance while considering age as a confounding factor of HRV [34], AHI, and BMI. Additionally, BMI was also independently related to non-REM-AHI and REM-AHI. Moreover, BMI z-score is not a good predictor of adiposity changes over time in children with obesity [35]. Therefore, actual BMI might serve as a reasonable marker of obesity in our children with OSA, evidenced by stronger associations between BMI and a range of variables, including demographic characteristics, polysomnographic parameters, HRV metrics, and systemic inflammatory markers, compared to those observed with BMI z-scores. Notably, the SDNN/SDSD ratio during REM sleep and age were both independently and positively associated with BMI, whereas the proportion of the REM stage was inversely related to BMI.

In our study, we discovered that the SDSD, an indicator of parasympathetic nerve activity [36], during N3 sleep was an independent variable of AHI. The balance between sympathetic and parasympathetic activities during N3 sleep influenced the relationship between BMI and IL-1RA levels in children with OSA, highlighting the influence of cardiac autonomic function on AHI, obesity, and IL-1RA levels. N3 sleep, a stage critical for glymphatic activity, which plays a major role in brain cleansing, is heavily influenced by the autonomic nervous system’s balance and the sleep-wake cycle [37]. Typically, parasympathetic nerve activity increases at the onset of sleep and reaches its peak during N3 sleep [38].

Parasympathetic nerve activation itself is not a direct cause of sleep apnea. Instead, the condition is related to inspiratory flow limitation or increased respiratory effort during sleep [39]. Furthermore, OSA is marked by pronounced sympathoexcitation, which can disrupt this balance [40]. Consequently, autonomic dysfunction, involving an imbalance between sympathetic and parasympathetic activity, can contribute to the condition’s complexity but is not a primary trigger. Additionally, an elevated sympathetic/parasympathetic ratio (SDNN/SDSD ratio) during N3 sleep may influence glymphatic function, thereby mediating the relationship between BMI and IL-1RA levels in pediatric patients. These findings underscore the complex interaction among sleep stages, sleep apnea, autonomic function, weight status, and anti-inflammatory regulation in children with OSA, highlighting the need to consider these elements comprehensively when managing the condition.

REM sleep is distinguished by rapid eye movements, electroencephalographic activity resembling wakefulness, muscle atonia, and vividly remembered dreams [41]. It is also marked by frequent changes in respiration and heart rate. Our results indicate that REM-AHI is higher than non-REM-AHI in children [42]. However, REM-AHI was not significantly associated with HRV metrics during the REM stage. Despite this, sympathetic nerve activation is more pronounced during apnea segments in patients with severe OSA [43]. Reduced vagal modulation during REM sleep has been frequently observed in previous research using HRV measurements [44]. In this study, we confirmed that pNN50, a parasympathetic modulation indicator [36], during REM sleep was inversely and independently related to AHI.

Importantly, reduced REM sleep has been independently linked to overweight children and adolescents [45]. There is a hypothesis suggesting that shorter durations of REM sleep may increase appetite due to elevated leptin levels [46]. In our study, we identified an increased SDNN/SDSD ratio (indicating sympathetic nerve activity relative to parasympathetic nerve activity) during REM sleep as an independent risk factor for reduced mean SpO_2_ and elevated BMI. Previous research suggests that a sympathovagal imbalance, characterized by reduced parasympathetic activity and/or increased sympathetic activity, may be secondary to sleep apnea in terms of increased AHI and reduced mean SpO_2_ [44] and contribute to the relationship between poor sleep (such as later bedtimes and sleep-disordered breathing) and obesity [47]. Thus, a higher autonomic imbalance during REM sleep and/or a lower percentage of REM sleep may contribute to an increase in OSA severity and BMI in children with OSA.

To address the complex intra-group relationships among variables, we conducted mediation and moderation analyses of BMI, polysomnographic parameters, HRV metrics, IL-1RA, and IL-6. These analyses identified IL-1RA levels as a mediator in the relationship between BMI and IL-6 levels, the SDNN/SDSD ratio during N3 sleep as a moderator between BMI and IL-1RA levels, and minimum SpO_2_ as a moderator between IL-1RA and IL-6 levels. This study underscored the complex interplay among BMI, sympathovagal balance during N3 sleep, minimum SpO_2_, IL-6, and IL-1RA in pediatric OSA, highlighting the critical importance of weight management in this demographic.

The accumulation of pro-inflammatory macrophages in adipose tissues is a key feature of obesity, which is recognized as a chronic inflammatory disease. This condition significantly contributes to the release of cytokines, thereby playing a pivotal role in the onset of metabolic complications associated with obesity [48]. IL-6, a pleiotropic inflammatory cytokine, is closely linked to obesity and functions as a “metabolic hormone”, influencing the homeostatic regulation of glucose, protein, and lipid metabolism [49]. Consequently, IL-6 serves as a critical inflammatory mediator in obesity, with its secretion influenced by a range of physiological or pathological factors, including hormones, cytokines, diet, physical activity, stress, and hypoxia [50]. In the context of obesity, there is an upregulation of IL-6 expression in adipose tissue, which exacerbates metabolic inflammation [51]. Therefore, in children with OSA, an increase in BMI coupled with a rise in circulatory IL-6 levels could serve as an early indicator of the potential development of metabolic disorders.

Circulatory IL-1RA acts as a naturally occurring antagonist of the IL-1 receptor, serving as an anti-inflammatory cytokine that helps modulate diabetogenesis [52]. In adults with obesity, serum concentrations of IL-1RA have been shown to be significantly elevated and are influenced by factors such as serum leptin levels and lean body mass. This suggests that the obesity-related increase in IL-1RA could contribute to central leptin resistance in patients with obesity [53]. Notably, IL-1RA has been found to correlate more strongly with obesity measures compared to other cytokines [54]. By blocking the IL-1 receptor Type I, IL-1RA significantly inhibits free fatty acids-mediated expression of IL-6 [55]. Furthermore, both IL-1RA and IL-6 have been positively associated with hypoxia in animal and human models [56,57], as well as measures of obesity and insulin resistance in nondiabetic adults [58].

Notably, reduced minimum SpO_2_ correlated with increased BMI and decreased the positive effect of IL-1RA levels on the IL-6 level in this study. Minimum SpO_2_ may bidirectionally modulate IL-6 levels in children with OSA. Nevertheless, while the precise mechanisms remain unclear, our findings suggest that increased levels of IL-1RA may mediate the association between BMI and IL-6, potentially moderating the metabolic effects of IL-6 by reducing susceptibility to diabetes mellitus [59].

Previous studies, primarily focusing on IL-6 and C-reactive peptide, have demonstrated that the tone of the parasympathetic nervous system, as inferred by HRV, is inversely related to inflammatory markers. HRV has been found to be inversely correlated with inflammatory markers both in healthy individuals and those with cardiovascular diseases [60]. Additionally, higher levels of IL-1RA have been associated with increased heart rates. A large cohort study observed associations between BMI, various HRV metrics (including SDNN, root mean square of successive differences between N-N intervals (RMSSD), total power, low-frequency power, and a low-frequency/high-frequency ratio), IL-1RA levels, and IL-6 levels, indicating that these HRV metrics reflect both sympathetic and parasympathetic nervous activities [61].

While our findings offer intriguing insights, this study is subject to several limitations that warrant mention. Firstly, a significant proportion (17%) of the 66 participants had no accessible polysomnography and HRV data, which further limited the sample size. Additionally, the present study did not assess jaw size and form, which are risk factors for pediatric OSA in individuals of Asian descent [62]. Consequently, the sample size was relatively small and confined to a single ethnic population, which may limit the generalizability of the results. Secondly, we did not assess the impacts of AHI in various stages on stage-specific HRV metrics in this study. Understanding these complex interactions across variables would benefit future studies. Thirdly, the cross-sectional nature of the study design precludes a definitive assessment of causality between the variables of interest. Consequently, there is a clear need for large-scale, longitudinal studies to further explore these relationships. Fourthly, the study population was homogeneous in ethnicity, which may limit the generalizability of the findings to other geographical locations and racial or ethnic backgrounds. Finally, the presence of chronic adenoiditis or tonsillitis in some participants could confound the analysis of systemic inflammation, potentially skewing the results. Despite these limitations, our study lays the groundwork for future research aimed at developing targeted therapeutic strategies to alleviate the disease burden in children with OSA.

## 4. Materials and Methods

### 4.1. Study Design and Participants

An observational and comparative study design was employed. Participants were prospectively recruited from the Department of Otolaryngology at the Linkou Main Branch of Chang Gung Memorial Hospital, Taoyuan, Taiwan, from 1 March 2017 to 31 January 2019. The Institutional Review Board of the Chang Gung Medical Foundation granted ethical approval for this research (Approval No.: 201507279A3). Written informed consent was obtained from both the parents (complete parent version) and the participants who were 6 years of age or older (brief children version). For participants younger than 6 years, only the parents’ signed complete parent version of the written informed consent was required. The study was conducted in compliance with the revised Declaration of Helsinki and the STROBE guidelines [63,64]. Figure 1 illustrates the selection process for participants in the study.

The study included children aged from ≥5.0 years old to 12.9 years old with an AHI of ≥5.0 events/hour, or an AHI of ≥2.0 events/hour accompanied by at least one morbidity, such as elevated blood pressure, daytime sleepiness, or growth retardation [65]. Exclusion criteria included children with craniofacial, neuromuscular, or chronic inflammatory disorders, such as asthma, atopic, or autoimmune diseases, were excluded [24,65]. Participants with acute inflammatory conditions or those requiring antibiotic therapy were only eligible for blood sample collection a minimum of 2 weeks post-remission [8]. Furthermore, participants without accessible sleep HRV data were excluded from the statistical analysis. Data collected included age, sex, tonsil size, ANR, and BMI [66]. Age and sex-adjusted BMI z-score was calculated using the United States Centers for Disease Control and Prevention 2000 growth charts [67].

### 4.2. Polysomnography

Standard in-laboratory polysomnography, utilizing equipment from Nicolet Biomedical Inc., Madison, WI, USA, was employed to evaluate pediatric OSA and measure various sleep parameters [24]. The AHI, AI, ArI, mean SpO_2_, minimum SpO_2_, total sleep time, and sleep stages, including N1, N2, N3, and REM stages, were assessed and validated manually by experienced investigators according to the American Academy of Sleep Medicine’s 2012 guidelines [68].

### 4.3. Sleep HRV Analysis

HRV analysis was performed on electrocardiographic signals obtained from polysomnography using the profusionSLEEP™ software (Version 4.5, build 502, Compumedics, Abbotsford, Australia). Experienced technicians manually inspected and verified automated annotations to identify artifacts, such as loose leads and motion artifacts, as well as arrhythmias [69]. For the primary analysis, all 2 min sleep epochs free from respiratory events or movement artifacts were selected. This approach was aimed at assessing persistent changes in HRV following respiratory events and establishing a more consistent baseline for HRV analysis [70,71]. HRV metrics were evaluated across different sleep stages, including N1, N2, N3, and REM. Time-domain HRV indices were calculated according to standard guidelines [70], including the SDNN, the SDSD, the SDNN/SDSD ratio, the pNN50, and the RMSSD. Notably, SDNN was utilized to assess sympathetic nerve activity, while SDSD, pNN50, and RMSSD were indicators of parasympathetic nerve activity [36]. Herein, the SDNN/SDSD ratio was calculated to quantify the balance between sympathetic and parasympathetic nerve activities. The Institutional Review Board of the Chang Gung Medical Foundation granted ethical approval for the analysis of HRV metrics (Approval No.: 202200882B0).

### 4.4. Systemic Inflammatory Biomarkers

Morning blood draws were conducted, with serum promptly isolated and stored at –80 °C pending analysis. Serum concentrations of IL-1β, IL-1RA, IL-6, and TNF-α were quantified using the Bio-Plex^®^ Pro Human Cytokine assay (Bio-Rad Laboratories, Hercules, CA, USA), in line with established protocols. Sample processing entailed centrifugation, dilution, and incubation with both antibody-coupled beads and detection antibodies, followed by streptavidin application as per the manufacturer’s recommendations and our previous report [24]. Quantification was performed using the Bio-Rad Bio-Plex Luminex 200 system with Bio-Plex Manager software (Version 6.0).

### 4.5. Sample-Size Estimation

The sample size was estimated using a primary outcome effect (sleep SDNN) based on data from a prior study (overweight/obesity group = 80.2 ± 30.5 and healthy weight group = 105.3 ± 33.7) [7]. We conducted a two-tailed independent-sample *t*-test to calculate the required sample size, with an effect size of 0.78, a Type I error of 0.05, and a power of 0.80. This calculation indicated that a total sample size of 54 participants would be sufficient.

### 4.6. Statistical Analysis

Data were analyzed using G*Power 3.1.9.2 (Heinrich–Heine University, Düsseldorf, Germany), SPSS Version 27.0 (IBM Corp., Armonk, NY, USA), and GraphPad Prism 10.0 for Windows (Graph Pad Software Inc., San Diego, CA, USA). Descriptive statistics included the mean (standard deviation) for continuous variables and frequency (proportion) for categorical variables. Differences in continuous variables between groups were evaluated using the independent-sample *t*-test. One-way MANOV with a Box’s test of equality of covariance matrices was used to determine differences between two groups in the proportions of the four sleep stages. Fisher’s exact test was employed for analyzing differences in categorical variables between two subgroups.

Correlations between variables of interest were assessed using Pearson and Point-Biserial correlation tests as appropriate. The Bonferroni correction was applied to reduce the Type I error due to multiple comparisons. Multivariable linear regression models of all variables were employed to identify independent predictors, utilizing forward variable selection based on a significance threshold of *F* < 0.05. The VIF was calculated for each predictor to adjust for intervariable relationships. To mitigate multicollinearity, regression models were recalibrated, excluding any variable with a VIF of 5 or higher.

Conditional process analysis, using the PROCESS macro (version 4.3) [72], assessed the mediators and moderators affecting the relationships between BMI and systemic inflammatory biomarkers. Mediation, moderated mediation, and mediated moderation were confirmed through bias-corrected 95% confidence intervals estimated via bootstrapping (5000 iterations). Statistical significance was determined by a two-sided *p*-value of <0.05.

## 5. Conclusions

Overall, our study provides a comprehensive analysis of the interactions between BMI, polysomnographic parameters, HRV, and inflammatory markers (IL-6 and IL-1RA) in pediatric patients with OSA, offering valuable insights into the complex underlying mechanisms. We have established that BMI serves as a reasonable indicator of obesity in these children, evidenced by significant associations with key variables of interest and improving the explanation of the variance. Importantly, our findings highlight the critical roles of minimum SpO_2_, as well as sympathetic and parasympathetic nerve activities during N3 and REM sleep stages in the development of OSA, obesity, and systemic inflammation. Disruptions in these sleep stages appear to contribute to systemic inflammation in children with OSA. This research sets the stage for future investigations to further elucidate these relationships through large-scale, longitudinal studies and to develop targeted therapeutic strategies that tackle the multifaceted nature of pediatric OSA. Such future research endeavors are essential to improve our understanding and management of OSA and obesity, potentially reducing its impact and enhancing outcomes for affected children. Our findings underscore the necessity of addressing obesity and autonomic imbalance as part of a comprehensive approach to managing pediatric OSA, which could significantly alter the progression and severity of associated inflammatory complications.

## Figures and Tables

**Table 1:** Participant demographic characteristics, polysomnographic parameters, and systemic inflammatory markers between overweight/obesity and healthy weight groups.

	Overall	Overweight/Obesity	Healthy Weight	*p*-Value ^1^
N (%)	55	30	25	
Demographic characteristics				
Age, years	7.4 ± 2.2	**8.0 ± 2.3**	**6.6 ± 1.8**	**0.01**
Sex, female/male	12/43	5/25	7/18	0.35
BMI, kg/m^2^	19.43 ± 5.55	**23.14 ± 4.81**	**14.98 ± 1.84**	**<0.01**
BMI z-score	0.680 ± 2.109	**1.995 ± 0.585**	**−0.900 ± 2.200**	**<0.01**
Tonsil size	3.2 ± 0.6	3.2 ± 0.5	3.3 ± 0.6	0.62
ANR	0.754 ± 0.130	0.724 ± 0.134	0.789 ± 0.119	0.07
Polysomnographic parameters				
AHI, events/hour	14.12 ± 17.05	**18.35 ± 18.36**	**9.05 ± 1.06**	**0.04**
Non-REM-AHI, events/hour	13.73 ± 18.05	16.19 ± 19.01	10.73 ± 16.71	0.29
REM-AHI, events/hour	21.35 ± 25.70	**29.13 ± 28.47**	**11.87 ± 18.34**	**0.01**
AI, events/hour	4.73 ± 8.89	5.11 ± 8.24	4.27 ± 0.24	0.73
ArI, events/hour	15.19 ± 16.67	**18.31 ± 13.52**	**11.58 ± 9.26**	**0.04**
Mean SpO_2_, %	97.2 ± 1.5	97.0 ± 0.91	97.5 ± 1.9	0.30
Minimum SpO_2_, %	88.0 ± 7.0	86.4 ± 5.7	89.9 ± 8.0	0.10
TST, minutes	331.1 ± 43.2	328.8 ± 46.7	337.2 ± 38.8	0.34
Sleep stages				
N1, %	13.1 ± 10.8	**16.1 ± 11.8**	**9.6 ± 8.5**	**0.0** **2**
N2, %	39.8 ± 8.0	39.3 ± 9.3	40.4 ± 6.2	0.61
N3, %	28.2 ± 8.8	27.1 ± 8.0	29.5 ± 9.8	0.33
REM, %	18.8 ± 5.9	17.5 ± 5.8	20.3 ± 5.7	0.08
Systemic inflammatory markers				
IL-1β, (pg/mL)	0.77 ± 0.85	0.61 ± 0.72	0.97 ± 0.97	0.11
IL-1RA, (pg/mL)	167.54 ± 130.11	182.22 ± 124.84	149.93 ± 136.62	0.36
IL-6, (pg/mL)	2.21 ± 4.00	2.62 ± 5.26	1.73 ± 1.43	0.42
TNF-α, (pg/mL)	42.91 ± 19.51	40.86 ± 20.84	45.37 ± 17.90	0.40

Data are summarized as mean ± standard deviation or *n* as appropriate. Abbreviations: AHI: apnea-hypopnea index; AI: apnea index; ANR: adenoidal-nasopharyngeal ratio; ArI: arousal index; BMI: body mass index; IL: interleukin; IL-1RA: IL-1 receptor antagonist; REM: rapid eye movement; SpO_2_: blood oxygen saturation; TNF: tumor necrosis factor; TST: total sleep time. ^1^ Differences in continuous and categorical variables between groups were evaluated using the independent-sample *t*-test or Fisher’s exact test as appropriate. Significant *p*-values are marked in bold.

**Table 2:** Heart-rate variability metrics across different sleep stages between overweight/obesity and healthy weight groups.

	Overall	Overweight/Obesity	Healthy Weight	*p*-Value ^1^
N (%)	55	30	25	
Stage N1 sleep				
N-N interval, ms	713.4 ± 100.0	721.2 ± 105.2	704.7 ± 95.6	0.67
SDNN, ms	59.17 ± 37.42	56.99 ± 37.64	61.56 ± 37.96	0.75
SDSD, ms	38.21 ± 30.02	37.76 ± 33.02	38.71 ± 27.16	0.93
SDNN/SDSD ratio	1.794 ± 0.568	1.853 ± 0.655	1.729 ± 0.461	0.40
pNN50, %	26.65 ± 23.30	25.60 ± 24.26	27.81 ± 22.75	0.80
RMSSD, ms	56.63 ± 42.74	55.43 ± 45.52	57.94 ± 40.54	0.86
Stage N2 sleep				
N-N interval, ms	735.1 ± 106.9	742.7 ± 117.4	726.8 ± 94.4	0.54
SDNN, ms	58.41 ± 34.27	56.10 ± 36.08	60.95 ± 32.87	0.64
SDSD, ms	39.95 ± 28.08	38.86 ± 30.55	41.13 ± 25.80	0.81
SDNN/SDSD ratio	1.688 ± 0.548	1.766 ± 0.693	1.603 ± 0.321	0.52
pNN50, %	33.68 ± 26.37	32.20 ± 27.77	35.31 ± 25.33	>0.99
RMSSD, ms	64.75 ± 44.47	63.11 ± 47.76	66.54 ± 41.68	0.87
Stage N3 sleep				
N-N interval, ms	747.3 ± 106.5	752.0 ± 121.6	742.2 ± 89.9	0.75
SDNN, ms	52.66 ± 32.85	51.90 ± 34.17	53.49 ± 32.17	0.81
SDSD, ms	36.57 ± 24.91	34.41 ± 25.93	36.75 ± 24.39	0.79
SDNN/SDSD ratio	1.578 ± 0.365	1.600 ± 0.431	1.553 ± 0.285	0.60
pNN50, %	35.06 ± 27.60	34.95 ± 27.43	35.17 ± 28.45	0.65
RMSSD, ms	62.44 ± 42.13	61.00 ± 41.73	64.02 ± 43.53	0.86
Stage REM sleep				
N-N interval, ms	711.7 ± 13.4	711.7 ± 93.7	711.8 ± 85.0	0.34
SDNN, ms	54.57 ± 43.67	49.97 ± 35.87	59.61 ± 11.20	0.71
SDSD, ms	37.69 ± 45.23	31.23 ± 30.51	44.77 ± 57.21	0.46
SDNN/SDSD ratio	2.019 ± 0.927	2.247 ± 1.111	1.769 ± 0.604	0.18
pNN50, %	20.91 ± 21.16	19.80 ± 20.52	22.11 ± 22.28	0.69
RMSSD, ms	53.74 ± 55.44	45.55 ± 41.32	62.71 ± 67.60	0.53

Data are summarized as mean ± standard deviation or *n* as appropriate. Abbreviations: N-N: normal-to-normal; pNN50: proportion of N-N interval pairs differing by more than 50 ms; REM: rapid eye movement; RMSSD: root mean square of successive differences between N-N intervals; SDNN: standard deviation of all N-N intervals; SDSD: standard deviation of successive differences between N-N intervals. ^1^ Differences in continuous variables between groups were evaluated using the independent-sample *t*-test.

## Data Availability

The data presented in this study are available on request from the corresponding author due to ethical reasons.

## Abbreviations

AHI	Apnea-hypopnea index
AI	Apnea index
ANR	Adenoidal-nasopharyngeal ratio
ArI	Arousal index
BMI	Body mass index
HRV	Heart rate variability
IL	Interleukin
IL-1RA	IL-1 receptor antagonist
MANOVA	Multivariate analysis of variance
NLRP3	Nucleotide-binding oligomerization domain-like receptor 3
N-N	Normal-to-normal
OSA	Obstructive sleep apnea
pNN50	Proportion of N-N interval pairs differing by more than 50 ms
REM	Rapid eye movement
RMSSD	Root mean square of successive differences between N-N intervals
SDNN	Standard deviation of all N-N intervals
SDSD	Standard deviation of successive differences between N-N intervals
SpO_2_	Peripheral oxygen saturation
TNF-α	Tumor necrosis factor-α
VIF	Variance inflation factor

## References

[1] Magnusdottir S., Hill E.A. (2024). Prevalence of obstructive sleep apnea (OSA) among preschool aged children in the general population: A systematic review. Sleep Med. Rev..

[2] Bitners A.C., Arens R. (2020). Evaluation and Management of Children with Obstructive Sleep Apnea Syndrome. Lung.

[3] Xu Z., Wu Y., Tai J., Feng G., Ge W., Zheng L., Zhou Z., Ni X. (2020). Risk factors of obstructive sleep apnea syndrome in children. J. Otolaryngol. Head. Neck Surg..

[4] Malhotra A., Ayappa I., Ayas N., Collop N., Kirsch D., McArdle N., Mehra R., Pack A.I., Punjabi N., White D.P. (2021). Metrics of sleep apnea severity: Beyond the apnea-hypopnea index. Sleep.

[5] Chuang H.H., Hsu J.F., Chuang L.P., Chen N.H., Huang Y.S., Li H.Y., Chen J.Y., Lee L.A., Huang C.G. (2020). Differences in Anthropometric and Clinical Features among Preschoolers, School-Age Children, and Adolescents with Obstructive Sleep Apnea-A Hospital-Based Study in Taiwan. Int. J. Env. Environ. Res. Public Health.

[6] de Araujo Lopes L.L., Costa F.W.G., Cevidanes L.H.S., de Barros Silva P.G., Gurgel M.L., Carvalho F.S.R., Junior C.M.C., Ribeiro T.R. (2024). Anthropometric measures and obstructive sleep apnea in children and adolescents: A systematic review of the literature and meta-analysis. Sleep Breath..

[7] Lee L.A., Chuang H.H., Hsieh H.S., Wang C.Y., Chuang L.P., Li H.Y., Fang T.J., Huang Y.S., Lee G.S., Yang A.C. (2023). Using sleep heart rate variability to investigate the sleep quality in children with obstructive sleep apnea. Front. Public Health.

[8] Huang C.G., Hsu J.F., Chuang L.P., Li H.Y., Fang T.J., Huang Y.S., Yang A.C., Lee G.S., Kuo T.B.J., Yang C.C.H. (2023). Adenotonsillectomy-related changes in systemic inflammation among children with obstructive sleep apnea. J. Chin. Med. Assoc..

[9] Hsieh H.S., Chuang H.H., Hsin L.J., Lin W.N., Kang C.J., Zhuo M.Y., Chuang L.P., Huang Y.S., Li H.Y., Fang T.J. (2023). Effect of Preoperative Weight Status and Disease Presentation on Postoperative Elevated Blood Pressure After Childhood Adenotonsillectomy. Otolaryngol.—Head Neck Surg..

[10] Li H.Y., Huang Y.S., Chen N.H., Fang T.J., Lee L.A. (2006). Impact of adenotonsillectomy on behavior in children with sleep-disordered breathing. Laryngoscope.

[11] Shetty M., Perera A., Kadar M., Tan B., Davey M.J., Nixon G.M., Walter L.M., Horne R.S. (2023). The effects of sleep disordered breathing on sleep spindle activity in children and the relationship with sleep, behavior and neurocognition. Sleep Med..

[12] Chen L.D., Chen M.X., Chen G.P., Lin X.J., Huang J.F., Zeng A.M., Huang Y.P., Lin Q.C. (2021). Association between obstructive sleep apnea and non-alcoholic fatty liver disease in pediatric patients: A meta-analysis. Pediatr. Obes..

[13] O’Driscoll D.M., Horne R.S., Davey M.J., Hope S.A., Anderson V., Trinder J., Walker A.M., Nixon G.M. (2011). Increased sympathetic activity in children with obstructive sleep apnea: Cardiovascular implications. Sleep Med..

[14] Campos J.O., Barros M.A.V., Oliveira T., Nobre I.G., de Morais A.S., Santos M.A.M., Leandro C.G., Costa-Silva J.H. (2022). Cardiac autonomic dysfunction in school age children with overweight and obesity. Nutr. Metab. Cardiovasc. Dis..

[15] Draghici A.E., Taylor J.A. (2016). The physiological basis and measurement of heart rate variability in humans. J. Physiol. Anthr. Anthropol..

[16] Drury R.L., Porges S., Thayer J., Ginsberg J.P. (2019). Editorial: Heart rate variability, health and well-being: A systems perspective. Front. Public Health.

[17] Siecinski S., Kostka P.S., Tkacz E.J. (2020). Heart rate variability analysis on electrocardiograms, seismocardiograms and gyrocardiograms on healthy volunteers. Sensors.

[18] Lopes M.C., Spruyt K., Azevedo-Soster L., Rosa A., Guilleminault C. (2019). Reduction in Parasympathetic Tone During Sleep in Children With Habitual Snoring. Front. Neurosci..

[19] Xie J.Y., Liu W.X., Ji L., Chen Z., Gao J.M., Chen W., Chen G.F., Zhu Q. (2020). Relationship between inflammatory factors and arrhythmia and heart rate variability in OSAS patients. Eur. Rev. Med. Pharmacol. Sci..

[20] Fitzpatrick S.F., King A.D., O’Donnell C., Roche H.M., Ryan S. (2021). Mechanisms of intermittent hypoxia-mediated macrophage activation—potential therapeutic targets for obstructive sleep apnoea. J. Sleep Res..

[21] Tang T., Huang Q., Liu J., Zhou X., Du J., Wu H., Li Z. (2019). Oxidative stress does not contribute to the release of proinflammatory cytokines through activating the Nod-like receptor protein 3 inflammasome in patients with obstructive sleep apnoea. Sleep Breath..

[22] Engin A. (2017). The Pathogenesis of Obesity-Associated Adipose Tissue Inflammation. Adv. Exp. Med. Biol..

[23] Taylor E.B. (2021). The complex role of adipokines in obesity, inflammation, and autoimmunity. Clin. Sci..

[24] Chuang H.H., Huang C.G., Chuang L.P., Huang Y.S., Chen N.H., Li H.Y., Fang T.J., Hsu J.F., Lai H.C., Chen J.Y. (2020). Relationships among and predictive values of obesity, inflammation markers, and disease severity in pediatric patients with obstructive sleep apnea before and after adenotonsillectomy. J. Clin. Med..

[25] Gaspar J.M., Velloso L.A. (2018). Hypoxia Inducible Factor as a Central Regulator of Metabolism—Implications for the Development of Obesity. Front. Neurosci..

[26] Prabhakar N.R., Peng Y.J., Nanduri J. (2020). Hypoxia-inducible factors and obstructive sleep apnea. J. Clin. Investig..

[27] Marseglia L., Manti S., D’Angelo G., Nicotera A., Parisi E., Di Rosa G., Gitto E., Arrigo T. (2014). Oxidative stress in obesity: A critical component in human diseases. Int. J. Mol. Sci..

[28] Williams A., Scharf S.M. (2007). Obstructive sleep apnea, cardiovascular disease, and inflammation--is NF-kappaB the key?. Sleep Breath..

[29] Vandanmagsar B., Youm Y.H., Ravussin A., Galgani J.E., Stadler K., Mynatt R.L., Ravussin E., Stephens J.M., Dixit V.D. (2011). The NLRP3 inflammasome instigates obesity-induced inflammation and insulin resistance. Nat. Med..

[30] Diaz-Garcia E., Garcia-Tovar S., Alfaro E., Jaureguizar A., Casitas R., Sanchez-Sanchez B., Zamarron E., Fernandez-Lahera J., Lopez-Collazo E., Cubillos-Zapata C. (2022). Inflammasome Activation: A Keystone of Proinflammatory Response in Obstructive Sleep Apnea. Am. J. Respir. Crit. Care Med..

[31] Hu Y., Dai S., Zhao L., Zhao L. (2024). Research progress on the improvement of cardiovascular diseases through the autonomic nervous system regulation of the NLRP3 inflammasome pathway. Front. Cardiovasc. Med..

[32] de Onis M., Onyango A.W., Borghi E., Siyam A., Nishida C., Siekmann J. (2007). Development of a WHO growth reference for school-aged children and adolescents. Bull. World Health Organ..

[33] Must A., Anderson S.E. (2006). Body mass index in children and adolescents: Considerations for population-based applications. Int. J. Obes..

[34] Bobkowski W., Stefaniak M.E., Krauze T., Gendera K., Wykretowicz A., Piskorski J., Guzik P. (2017). Measures of Heart Rate Variability in 24-h ECGs Depend on Age but Not Gender of Healthy Children. Front. Physiol..

[35] Vanderwall C., Eickhoff J., Randall Clark R., Carrel A.L. (2018). BMI z-score in obese children is a poor predictor of adiposity changes over time. BMC Pediatr..

[36] Xiao B., Yin P.F., Jin Y.H., Liu F., Lu J.C., Yang X.C. (2022). Trimetazidine Effect on the Cardiac Autonomic Nerve System after Percutaneous Coronary Intervention in Coronary Heart Disease: A Propensity-score Matched Study. J. Coll. Physicians Surg. Pak..

[37] Nycz B., Mandera M. (2021). The features of the glymphatic system. Auton. Neurosci..

[38] Stein P.K., Pu Y. (2012). Heart rate variability, sleep and sleep disorders. Sleep Med. Rev..

[39] Lin C., Lo M.T., Guilleminault C. (2017). Exploring the Abnormal Modulation of the Autonomic Systems during Nasal Flow Limitation in Upper Airway Resistance Syndrome by Hilbert-Huang Transform. Front. Med..

[40] Weiss J.W., Tamisier R., Liu Y. (2015). Sympathoexcitation and arterial hypertension associated with obstructive sleep apnea and cyclic intermittent hypoxia. J. Appl. Physiol..

[41] Fraigne J.J., Torontali Z.A., Snow M.B., Peever J.H. (2015). REM Sleep at its Core—Circuits, Neurotransmitters, and Pathophysiology. Front. Neurol..

[42] Goh D.Y., Galster P., Marcus C.L. (2000). Sleep architecture and respiratory disturbances in children with obstructive sleep apnea. Am. J. Respir. Crit. Care Med..

[43] Romero D., Jane R. (2021). Relationship between Sleep Stages and HRV response in Obstructive Sleep Apnea Patients. Annu. Int. Conf. IEEE Eng. Med. Biol. Soc..

[44] Dissanayake H.U., Bin Y.S., Ucak S., de Chazal P., Sutherland K., Cistulli P.A. (2021). Association between autonomic function and obstructive sleep apnea: A systematic review. Sleep Med. Rev..

[45] Liu X., Forbes E.E., Ryan N.D., Rofey D., Hannon T.S., Dahl R.E. (2008). Rapid eye movement sleep in relation to overweight in children and adolescents. Arch. Gen. Psychiatry.

[46] Olson C.A., Hamilton N.A., Somers V.K. (2016). Percentage of REM sleep is associated with overnight change in leptin. J. Sleep Res..

[47] Jarrin D.C., McGrath J.J., Poirier P., Quality Cohort Collaborative G. (2015). Autonomic dysfunction: A possible pathophysiological pathway underlying the association between sleep and obesity in children at-risk for obesity. J. Youth Adolesc..

[48] Kriebs A. (2021). Accumulation of macrophages in adipose tissue. Nat. Rev. Endocrinol..

[49] Hunter C.A., Jones S.A. (2015). IL-6 as a keystone cytokine in health and disease. Nat. Immunol..

[50] Eder K., Baffy N., Falus A., Fulop A.K. (2009). The major inflammatory mediator interleukin-6 and obesity. Inflamm. Res..

[51] Sindhu S., Thomas R., Shihab P., Sriraman D., Behbehani K., Ahmad R. (2015). Obesity Is a Positive Modulator of IL-6R and IL-6 Expression in the Subcutaneous Adipose Tissue: Significance for Metabolic Inflammation. PLoS ONE.

[52] Volarevic V., Al-Qahtani A., Arsenijevic N., Pajovic S., Lukic M.L. (2010). Interleukin-1 receptor antagonist (IL-1Ra) and IL-1Ra producing mesenchymal stem cells as modulators of diabetogenesis. Autoimmunity.

[53] Meier C.A., Bobbioni E., Gabay C., Assimacopoulos-Jeannet F., Golay A., Dayer J.M. (2002). IL-1 receptor antagonist serum levels are increased in human obesity: A possible link to the resistance to leptin?. J. Clin. Endocrinol. Metab..

[54] Juge-Aubry C.E., Henrichot E., Meier C.A. (2005). Adipose tissue: A regulator of inflammation. Best Pr. Pract. Res. Clin. Endocrinol. Metab..

[55] Boni-Schnetzler M., Boller S., Debray S., Bouzakri K., Meier D.T., Prazak R., Kerr-Conte J., Pattou F., Ehses J.A., Schuit F.C. (2009). Free fatty acids induce a proinflammatory response in islets via the abundantly expressed interleukin-1 receptor I. Endocrinology.

[56] Hagberg H., Gilland E., Bona E., Hanson L.A., Hahin-Zoric M., Blennow M., Holst M., McRae A., Soder O. (1996). Enhanced expression of interleukin (IL)-1 and IL-6 messenger RNA and bioactive protein after hypoxia-ischemia in neonatal rats. Pediatr. Res..

[57] Andersson N., Strandberg L., Nilsson S., Ljungren O., Karlsson M.K., Mellstrom D., Lorentzon M., Ohlsson C., Jansson J.O. (2009). Variants of the interleukin-1 receptor antagonist gene are associated with fat mass in men. Int. J. Obes..

[58] Charles B.A., Doumatey A., Huang H., Zhou J., Chen G., Shriner D., Adeyemo A., Rotimi C.N. (2011). The roles of IL-6, IL-10, and IL-1RA in obesity and insulin resistance in African-Americans. J. Clin. Endocrinol. Metab..

[59] Perrier S., Darakhshan F., Hajduch E. (2006). IL-1 receptor antagonist in metabolic diseases: Dr Jekyll or Mr Hyde?. FEBS Lett..

[60] Haensel A., Mills P.J., Nelesen R.A., Ziegler M.G., Dimsdale J.E. (2008). The relationship between heart rate variability and inflammatory markers in cardiovascular diseases. Psychoneuroendocrinology.

[61] Hansen C.S., Vistisen D., Jorgensen M.E., Witte D.R., Brunner E.J., Tabak A.G., Kivimaki M., Roden M., Malik M., Herder C. (2017). Adiponectin, biomarkers of inflammation and changes in cardiac autonomic function: Whitehall II study. Cardiovasc. Diabetol..

[62] Kim C.Y., Reinertsen E., Dang C., Nkutshweu D., Sathekge R., Choi Y.J., Cha J.Y., Alturki G., Jamel A., Suzuki A. (2024). Association among craniofacial morphology, ethnicity, and risk of pediatric sleep-related breathing disorders: A multicenter study. Am. J. Orthod. Dentofac. Orthop..

[63] World Medical A. (2013). World Medical Association Declaration of Helsinki: Ethical principles for medical research involving human subjects. J. Am. Med. Assoc..

[64] von Elm E., Altman D.G., Egger M., Pocock S.J., Gotzsche P.C., Vandenbroucke J.P., Initiative S. (2007). The Strengthening the Reporting of Observational Studies in Epidemiology (STROBE) statement: Guidelines for reporting observational studies. PLoS Med..

[65] Chuang H.H., Hsu J.F., Chuang L.P., Chiu C.H., Huang Y.L., Li H.Y., Chen N.H., Huang Y.S., Chuang C.W., Huang C.G. (2021). Different associations between tonsil microbiome, chronic tonsillitis, and intermittent hypoxemia among obstructive sleep apnea children of different weight status: A pilot case-control Study. J. Pers. Med..

[66] Brodsky L. (1989). Modern Assessment of Tonsils and Adenoids. Pediatr. Clin. North. Am..

[67] Flegal K.M., Cole T.J. (2013). Construction of LMS parameters for the Centers for Disease Control and Prevention 2000 growth charts. Natl. Health Stat. Rep..

[68] Berry R.B., Budhiraja R., Gottlieb D.J., Gozal D., Iber C., Kapur V.K., Marcus C.L., Mehra R., Parthasarathy S., Quan S.F. (2012). Rules for scoring respiratory events in sleep: Update of the 2007 AASM Manual for the Scoring of Sleep and Associated Events. Deliberations of the Sleep Apnea Definitions Task Force of the American Academy of Sleep Medicine. J. Clin. Sleep Med..

[69] Littmann L. (2021). Electrocardiographic artifact. J. Electrocardiol..

[70] Malik M., Task Force of the European Society of Cardiology and the North American Society of Pacing and Electrophysiology (1996). Heart rate variability: Standards of measurement, physiological interpretation and clinical use. Circulation.

[71] Muzumdar H.V., Sin S., Nikova M., Gates G., Kim D., Arens R. (2011). Changes in heart rate variability after adenotonsillectomy in children with obstructive sleep apnea. Chest.

[72] Hayes A.F. (2022). Introduction to Mediation, Moderation and Conditional Process Analysis: A Regression-Based Approach.

